# Use of chloroquine, hydroxychloroquine or ivermectin for Covid-19 prevention in vulnerable Brazilian populations

**DOI:** 10.11606/s1518-8787.2026060006988

**Published:** 2026-05-01

**Authors:** Débora Castanheira, Thiago Silva Torres, Thais Regis Aranha, Fabiane Soares, Ines Dourado, Valdilea Gonçalves Veloso

**Affiliations:** I Fundação Oswaldo Cruz. Instituto Nacional de Infectologia Evandro Chagas. Rio de Janeiro, RJ, Brasil; II Universidade Federal da Bahia. Instituto de Saúde Coletiva. Salvador, BA, Brasil

**Keywords:** Chloroquine, Hydroxychloroquine, Ivermectin, COVID-19, Communication

## Abstract

**OBJECTIVE:**

To explore factors associated with the use of chloroquine, hydroxychloroquine, and ivermectin for Covid-19 prevention in socioeconomically vulnerable populations in Brazil.

**METHODS:**

A cross-sectional study was conducted using data from the project "Expansion of testing, quarantine, digital health, and telemonitoring strategies to tackle the Covid-19 pandemic in Brazil." Participants were users of 19 primary healthcare units in Salvador (Bahia, BA) and Rio de Janeiro (Rio de Janeiro, RJ) from July 2022 to July 2023. Data were collected via a socioeconomic questionnaire and analyzed using logistic regression to assess factors associated with the use of chloroquine, hydroxychloroquine, or ivermectin for Covid-19 prevention. Multicollinearity was assessed using the generalized variance inflation factor (GVIF), with GVIF^(1/(2*df)) > 5 indicating potential collinearity. Sensitivity analyses were performed using the same backward selection procedure as the main model: excluding "sometimes" responses and stratifying analyses by city (Rio de Janeiro and Salvador).

**RESULTS:**

Among 7,505 participants, 11.7% reported using chloroquine, hydroxychloroquine, or ivermectin for Covid-19 prevention. Use was more frequent among people who identified themselves as Brown (OR_a_ = 1.38; 95%CI 1.10–1.75), aged 35–44 (OR_a_ = 1.34; 95%CI 1.03–1.75) or 44–59 (OR_a_ = 1.36; 95%CI 1.06–1.77), evangelical (OR_a_ = 1.32; 95%CI 1.14–1.53), and with comorbidities (OR_a_ = 1.25; 95%CI 1.07–1.47). Having up to two doses of Covid-19 vaccine (OR_a_ = 1.30; 95%CI 1.06–1.59) and being unvaccinated while living with someone with comorbidities (OR_a_ = 10.34; 95%CI 2.27–53.48) also increased the odds of use. GVIF values were low except for city (8.79), due to its interaction with income; the variable was retained for conceptual reasons. Sensitivity analyses yielded results consistent with the main model.

**CONCLUSION:**

The use of ineffective medications for Covid-19 prevention was higher among specific demographic groups, reflecting inequalities in access to information and the influence of religious factors. Scientific communication and community engagement strategies remain essential to combat misinformation.

## INTRODUCTION

At the onset of the Covid-19 pandemic, chloroquine, hydroxychloroquine, and ivermectin were widely used to prevent Covid-19 without solid evidence of their efficay^1-4^. These drugs were broadly distributed in Latin America to treat mild Covid-19 cases^1^ and prevent infections^3,4^.

Chloroquine and hydroxychloroquine, traditionally used for malaria and rheumatic diseases^2^, were initially adopted for Covid-19 treatment in many countries^5^. However, early clinical trials showed that chloroquine did not affect clinical outcomes or mortality, leading to its discontinued use^2^. In China and South Korea, for example, these drugs were abandoned after randomized controlled trials and systematic reviews demonstrated no preventive effect and highlighted safety concerns, prompting regulatory restrictions^6^. Similar findings led regulatory agencies in Europe to restrict their use outside clinical trials due to safety and efficacy issues. In the United States (US), the Food and Drug Administration (FDA) approved an emergency use authorization at the beginning of the pandemic, though it was later revoked. Chloroquine and hydroxychloroquine, however, continued to be widely used for Covid-19 prevention, being promoted by US political leaders and incorporated into some local treatment protocols^7^. Inconsistent data on ivermectin efficacy led major health authorities, including the World Health Organization (WHO) and National Institutes of Health (NIH), to advise against its routine use outside clinical research. Nonetheless, ivermectin use also surged in the US, fueled largely by social media, political endorsement, and advocacy from some professional groups^8^.

In Brazil, recommendations for chloroquine and hydroxychloroquine persisted longer due to the federal government's stance against scientific evidence^4,9^. Ivermectin, an antiparasitic drug, was also adopted based on *in vitro* studies suggesting potential antiviral properties^1^. Despite early evidence showing that ivermectin did not improve clinical outcomes^10^, it continued to be used in Brazil^1,9^.

These drugs were recommended by prescribers, health departments, and the federal government as part of early Covid-19 treatment and for prevention^3,4^, even though their indiscriminate use can cause significant health problems. Hydroxychloroquine and chloroquine can cause serious cardiac arrhythmias^2^, and common side effects of ivermectin include headaches, muscle pain, and gastrointestinal issues, with rare cases of severe inflammatory responses^1^. Overuse of these drugs can lead to drug-resistant pathogens^9^ and undermine public trust in health services^1^. Indeed, a study with 3,046 adults conducted in Manaus, Brazil, from August 2020 to October 2020, found that the seroprevalence of SARS-CoV-2 was higher among those who self-medicated to prevent Covid-19^3^.

The indiscriminate use of chloroquine, hydroxychloroquine, and ivermectin for Covid-19 has led to significant public health costs, including increased morbidity, mortality, and healthcare burden. Studies have shown that hydroxychloroquine use may be associated with harmful effects, such as increased mortality, higher rates of intensive care admission, and mechanical ventilation needs among Covid-19 patients, suggesting detrimental impacts when used outside controlled clinical settings^11,12^. Similarly, ivermectin has not demonstrated clear clinical benefits in reducing Covid-19 severity or mortality, and widespread off-label use fueled by misinformation and political endorsement has led to public health risks and resource misallocation^4,13^.

While some studies have explored self-medication for Covid-19^14-18^, evidence on the factors influencing the use of chloroquine, hydroxychloroquine, and ivermectin remains limited, particularly in Brazil^19,20^. This is critical for populations under socioeconomic vulnerability, which may be more prone to misinformation and self-medication with ineffective treatments^4,9^. The aim of this study was to explore the factors associated with the use of chloroquine, hydroxychloroquine, and ivermectin to prevent Covid-19 in populations under high socioeconomic vulnerability in Brazil.

## METHODS

### Study Design

This cross-sectional study used data from the project "Expansion of testing, quarantine, digital health and telemonitoring strategies to tackle the Covid-19 pandemic in Brazil", which included users of 19 public primary healthcare services (PHC) units located in neighborhoods under high socioeconomic vulnerability in Salvador (Bahia, BA) and Rio de Janeiro (Rio de Janeiro, RJ)^21^. A convenience sample was recruited between July 2022 and July 2023, with data collection occurring for six months in each PHC unit. Covid-19 testing was offered to individuals meeting the inclusion criteria: being a user of a PHC located in Cabula-Beiru (Salvador) and Manguinhos (Rio de Janeiro) health districts; presenting symptoms (3-7 days before testing) consistent with Covid-19 infection, or having been in close contact with a person diagnosed with Covid-19 (for asymptomatic individuals, testing was indicated if the contact occurred 3-7 days before testing). Exclusion criteria were active nosebleed, traumatic or other acute facial injury, or inability to provide informed consent. A total of 12,883 individuals were tested, of whom 8,633 answered the socio-behavioral questionnaire. Among these, 7,515 were aged ≥ 18 years, and 7,505 responded to the question regarding the use of chloroquine, hydroxychloroquine, or ivermectin for Covid-19 prevention; these individuals were included in the present analysis.

### Statistical Analysis

The main outcome was assessed through the question: "Which of these behaviors are currently part of your daily routine to prevent Covid-19: taking hydroxychloroquine, chloroquine, or ivermectin?", with response options "yes," "no," and "sometimes." For the primary analysis, responses "yes" and "sometimes" were grouped to indicate use of these medications for Covid-19 prevention.

Independent variables included in the multivariable model comprised sociodemographic and health-related characteristics. Sociodemographic variables were: city (open-ended field); age range, calculated from the "date of birth" and "date of questionnaire completion" questions (18-24 years, 25-34 years, 35-44 years, 44-59 years, and ≥ 60 years); gender identity, based on the question "How do you identify yourself?" (cisgender woman, cisgender man, *travesti*/trans woman, trans man, or other); race/skin color, from the question "What is your race/skin color?" (White, Brown, Black, Asian [Yellow], Indigenous); educational level, based on "What is the highest level of education you have attended?" (from no formal schooling to doctoral degree); income, from the question "Summing all salaries in your family, what is your household's monthly income?" (< 1, 1-2, > 2-5, > 5-10, > 10 minimum wages, or prefer not to answer); and religion, from the question "What is your religion?" (multiple responses allowed: no religion, evangelical protestant, *umbanda*, *candomblé*, kardecist spiritism, catholic, or other). Because multiple answers were possible for religion, a binary variable was created for evangelical affiliation (yes *versus* no) to ensure statistical model stability.

Health-related variables included: presence of at least one comorbidity, from the question "Have you ever been told by a doctor or other health professional that you have any of the following conditions?" allowing multiple responses from a list of chronic diseases (*e.g.*, obesity, *diabetes mellitus*, heart disease or hypertension, respiratory disease, cancer, hematologic disease, chronic kidney disease, chromosomal diseases with immunological fragility, liver disease, autoimmune diseases, immunodeficiencies, none, or other), and classified as yes/no; living with an older adult, from the question "Do you live with someone aged over 60 years?" (yes/no); living with someone with comorbidities, based on a similar multi-response list as the comorbidity question, also classified as yes/no; Covid-19 vaccination status, from the questions "Have you been vaccinated against Covid-19?" (yes/no) and "number of doses" (1 dose, 2 doses, single dose, first booster, second booster), recoded as unvaccinated, ≤ 2 doses, or > 2 doses; and elderly status (yes/no), derived from the age variable. All variables were treated as categorical factors in the analysis.

Bivariate analysis and binary logistic regression were used to explore factors associated with this outcome. Crude odds ratios (OR) were calculated in the bivariate analysis, and adjusted odds ratios (OR_a_) with 95% confidence intervals (95%CI) were estimated in the final multivariable model. All analyses were conducted using R (version 4.4.1).

The multivariable model initially included all theoretically relevant covariates: city, age group, gender identity, educational level, race/skin color, income, presence of at least one comorbidity, living with someone with comorbidities, Covid-19 vaccination status (unvaccinated, ≤ 2 or > 2 doses), and religious affiliation. Because the questionnaire allowed multiple responses for religion, participants could report belonging to more than one religious group, which precluded direct inclusion of religion as a multi-category factor in regression models. To address this and ensure model stability, a binary variable for evangelical affiliation (yes *versus* no) was created, selected due to its high prevalence and potential relevance to health communication and preventive behaviors in the study context. All variables were treated as categorical factors.

Multicollinearity was assessed using the generalized variance inflation factor (GVIF), which accounts for categorical predictors with multiple degrees of freedom. A threshold of GVIF^(1/(2*df)) > 5 was used to indicate potential multicollinearity. Model fit was evaluated using the Akaike information criterion (AIC). Decisions to retain or exclude variables in the final model were based on a combination of GVIF values, AIC comparisons, and theoretical relevance.

All two-way interaction terms between independent variables were systematically tested. Three interaction terms significantly improved model fit (p < 0.05 and ΔAIC > 2) and were included: city × income, vaccination status × living with someone with comorbidities, and vaccination status × education. Final model selection was performed using stepwise backward elimination based on AIC.

Missing data were minimal (< 0.5% for all variables), except for gender identity (0.51%). All participants with missing data on the dependent variable (n = 39) were excluded prior to analysis. Cases in which the question included the response option "Did not wish to answer" were treated as a response category rather than as missing data. Complete-case analysis was then applied for the independent variables. Categories with few observations (*e.g.*, non-binary gender identities) were retained, and wide confidence intervals were reported to reflect this uncertainty.

Sensitivity analyses were performed to assess the robustness of findings, using the same backward selection procedure as in the main model to ensure comparability. First, individuals who responded "sometimes" were excluded from the outcome variable to compare only "yes" *versus* "no" responses. Second, models were stratified by city (Rio de Janeiro and Salvador) to explore contextual differences in associations.

### Ethical Considerations

The study was approved by the WHO (protocol identification: #CERC.0128A and #CERC.0128B) and local ethical review boards (ISC/UFBA: #53844121.1001.5030, INI/Fiocruz: #53844121.4.3001.5240, ENSP/Fiocruz: #53844121.4.3001.5240, and SMS/RJ: #53844121.4.3002.5279).

## RESULTS

A total of 7,505 participants were included in the analysis. Most resided in Salvador (59.7%), identified as women (66.9%), and self-reported as Brown (48.6%) or Black (35.4%). The largest age group was 44-59 years (27.5%), followed by 25-34 years (23.8%). Nearly half had completed high school (43.9%), and 27.7% reported an income between 1 and 2 minimum wages. Evangelical Christians comprised 36.0% of the sample, while 15.7% reported no religion. Comorbidities were present in 42.8% of participants, and 31.3% lived with someone with a comorbidity. Most had received more than two Covid-19 vaccine doses (85.4%) (Table 1).

**Table 1:** Socio-demographic characteristics of respondents.

Socio-demographic characteristics		n	%	95%CI
Total		7,505	100	
City				
	Rio de Janeiro	1,954	26.04	25.05-27.05
	Salvador	5,551	73.96	72.95-74.95
Gender^a^				
	Women (cis and trans)	5,227	69.65	68.59-70.68
	Men (cis and trans)	2,226	29.66	28.63-30.71
	Non-binary	14	0.19	0.10-0.31
	Missing	38	0.51	0.36-0.69
Race/skin color				
	White	1,101	14.67	13.88-15.49
	Black	2,655	35.38	34.29-36.47
	Brown	3,650	48.63	47.50-49.77
	Asian	73	0.97	0.76-1.22
	Indigenous	19	0.25	0.15-0.40
	Missing	7	0.09	0.04-0.19
Age group (years)				
	18-24	1,005	13.39	12.63-14.18
	25-34	1,789	23.84	22.88-24.82
	35-44	1,605	21.39	20.46-22.33
	44-59	2,065	27.51	26.51-28.54
	≥ 60	1,041	13.87	13.10-14.67
Educational level				
	Higher education or above	1,565	20.85	19.94-21.79
	High school	3,294	43.89	42.76-45.02
	Primary school	1,674	22.31	21.37-23.26
	No formal education	74	0.99	0.78-1.24
	Did not wish to answer	898	11.97	11.24-12.72
Income (minimum wages)				
	More than 10	72	0.96	0.75-1.21
	More than 5 to 10	197	2.62	2.28-3.01
	More than 2 to 5	1,088	14.5	13.71-15.31
	From 1 to 2	2,077	27.67	26.66-28.70
	Less than 1	1,333	17.76	16.90-18.65
	Did not wish to answer	2,738	36.48	35.39-37.58
Religion^b^				
	No religion	1,179	15.71	14.90-16.56
	Evangelical Christian	2,699	35.96	34.88-37.06
	Catholic	2,741	36.52	35.43-37.62
	Afro-Brazilian religions	249	3.32	2.93-3.75
	Other religions	624	8.31	7.70-8.97
	Multiple religions	27	0.36	0.24-0.53

1 95%CI: 95% confidence interval.

a One trans woman was included.

b Participants could choose more than one religion.

Overall, 11.7% of participants reported using chloroquine, hydroxychloroquine, or ivermectin to prevent Covid-19. Prevalence was higher in Salvador (12.26%) than in Rio de Janeiro (10.31%). By gender, use was 11.42% among women, 11.91% among men, and 7.14% among other genders. Regarding race/skin color, prevalence was 9.39% for White persons, 11.09% for Black, 12.99% for Brown, 10.96% for Asians, and 5.26% for Indigenous participants. By age group, prevalence ranged from 9.4% among those aged 18-24 years to 13.15% in participants aged 44-59 years. Prevalence by educational level was similar across groups, ranging from 10.93% among those with primary school education to 13.51% among those with no formal education. In relation to income, use ranged from 9.4% among participants earning more than 10 minimum wages to 14.36% among those earning 5-10 minimum wages. Among religious groups, prevalence was highest among Evangelical Christians (13.82%) compared to 10.58% among those with no religion or other religions. Participants with comorbidities reported higher prevalence (13.21%) than those without (10.88%). Those vaccinated with ≤ 2 doses had higher prevalence (14.06%) than those with more than two doses (11.09%), while unvaccinated participants had the highest prevalence (16.67%) (Table 2).

**Table 2:** Prevalence of use of chloroquine, hydroxychloroquine, and ivermectin to prevent Covid-19 by sociodemographic and clinical characteristics.

Sociodemographic and clinical characteristics		Yes		No	
		n	%	n	%
Total		877	11.75	6,589	88.25
City					
	Rio de Janeiro	201	10.31	1,749	89.69
	Salvador	676	12.26	4,840	87.74
Gender					
	Women (cis and trans)	254	11.42	1,971	88.58
	Men (cis and trans)	622	11.9	4,605	88.1
	Other	1	7.14	13	92.86
Race/skin color					
	White	103	9.39	994	90.61
	Black	293	11.09	2,350	88.91
	Brown	472	12.99	3,162	87.01
	Asian	8	10.96	65	89.04
	Indigenous	1	5.26	18	94.74
Age group (years)					
	18-24	94	9.4	906	90.6
	25-34	199	11.19	1,580	88.81
	35-44	202	12.66	1,394	87.34
	44-59	270	13.15	1,784	86.85
	≥ 60	112	10.8	925	89.2
Educational level					
	Higher education or above	199	12.81	1,355	87.19
	High school	378	11.51	2,906	88.49
	Primary school	182	10.93	1,483	89.07
	No formal education	10	13.51	64	86.49
	Did not wish to answer	108	12.15	781	87.85
Income (minimum wages)^a^					
	More than 10	9	12.5	63	87.5
	More than 5 to 10	28	14.36	167	85.64
	More than 2 to 5	138	12.78	942	87.22
	From 1 to 2	257	12.41	1,814	87.59
	Less than 1	152	11.43	1,178	88.57
	Did not wish to answer	293	10.78	2,425	89.22
Religion^b^					
	Other religions or no religion	506	10.58	4,275	89.42
	Evangelical Christian	371	13.82	2,314	86.18
Comorbidity^c^					
	No	509	10.88	4,171	89.12
	Yes	368	13.21	2,418	86.79
Living with someone with comorbidity^c^					
	No	593	11.33	4,639	88.67
	Yes	284	12.71	1,950	87.29
Living with someone > 60 years					
	No	768	11.65	5,826	88.35
	Yes	109	12.5	763	87.5
Covid-19 vaccination status					
	More than two vaccine doses	652	11.09	5,225	88.91
	Up to two vaccine doses	215	14.06	1,314	85.94
	Unvaccinated	10	16.67	50	83.33

a Referring to the Brazilian minimum monthly wage in force in 2022 of R$ 1,212.00 or US$ 233.53 (values based on exchange rate on 12/01/2022).

b Participants could choose more than one religion;

c Obesity, *diabetes mellitus*, heart disease, high blood pressure, respiratory disease, cancer, hematological disease, chronic kidney disease at an advanced stage, chromosomal disorders with immune fragility, liver disease, autoimmune diseases or immunodeficiencies.

GVIF values were calculated to assess multicollinearity. Most variables had adjusted GVIF^(1/(2*df)) values below 2, indicating low collinearity. The highest values were observed for "city" (8.79) and the "city:income" interaction (2.61), with the elevated GVIF for "city" largely attributable to this interaction. However, excluding city worsened model fit based on the AIC. Given its conceptual importance in reflecting geographic variation in health communication and public policy, city was retained in the final model. This decision, despite elevated GVIF values, is acknowledged as a methodological limitation (Table 3).

**Table 3:** Assessment of multicollinearity using generalized variance inflation factor in the regression model.

Variable	GVIF	Df	GVIF^(1/(2*Df))
City	77.42	1	8.79
Income	572.80	5	1.89
Living with someone with comorbidity	1.45	1	1.20
Covid-19 vaccination status	2.64	2	1.27
Gender	1.04	2	1.01
Race/skin color	1.12	4	1.01
Comorbidity	1.06	1	1.03
Living with someone > 60 years	1.12	1	1.06
Religion­_binary^a^	1.03	1	1.02
Educational level	1.02	4	1.00
City:income	1,463.24	5	2.61
Living with someone with comorbidity: Covid-19 vaccination status	3.08	2	1.33

Df: degrees of freedom for each variable in the model (equal to 1 for continuous variables; for categorical variables, Df = number of categories - 1; for interaction terms, Df equals the product of the degrees of freedom of the factors involved); GVIF_1/(2×Df)_: adjusted Generalized Variance Inflation Factor, which allows a comparison across variables with different numbers of degrees of freedom;

a Evangelical Christian x any other religion or no religion.

Table 4 presents both crude and adjusted odds ratios (OR_a_) for the association between sociodemographic and clinical characteristics and the use of chloroquine, hydroxychloroquine, and ivermectin to prevent Covid-19. In the adjusted model, higher odds of use were observed among individuals identifying as Brown (OR_a_ = 1.38; 95%CI 1.10-1.75), those aged 35-44 years (OR_a_ = 1.34; 95%CI 1.03-1.75) and 44-59 years (OR_a_ = 1.36; 95%CI 1.06-1.77), evangelical Christians (OR_a_ = 1.32; 95%CI 1.14-1.53), and participants with comorbidities (OR_a_ = 1.25; 95%CI 1.07-1.47). Having up to two Covid-19 vaccine doses was also associated with higher odds of use (OR_a_ = 1.30; 95%CI 1.06-1.59). A strong association was found for unvaccinated individuals living with someone with comorbidities (OR_a_ = 10.34; 95%CI 2.27-53.48).

**Table 4:** Logistic regression model of the use of chloroquine, hydroxychloroquine, and ivermectin to prevent Covid-19, crude odds ratio and adjusted odds ratio.

Socio demographic and clinical characteristics		p-value	OR	95%CI	p-value	Adjusted OR	
			OR			OR	95%CI
City							
	Rio de Janeiro		1.00			1.00	
	**Salvador**	**0.02**	**1.22**	**0.1-0.13**	**0.39**	**1.90**	**0.46-9.69**
Gender							
	Women (cis and trans)		1.00		-	-	-
	Men (cis and trans)	0.82	0.95	0.82-1.11	-	-	-
	Other	0.59	0.57	0.03-2.87	-	-	-
Race/skin color							
	White		1.00			1.00	
	Black	0.13	1.20	0.95-1.53	0.38	1.12	0.87-1.44
	**Brown**	**0.00**	**1.44**	**1.16-1.81**	**0.01**	**1.38**	**1.10-1.75**
	Asian	0.66	1.19	0.51-2.41	0.76	1.13	0.49-2.30
	Indigenous	0.55	0.54	0.03-2.64	0.54	0.56	0.03-2.71
Age group (years)							
	18-24		1.00			1.00	
	25-34	0.14	1.21	0.94-1.58	0.23	1.17	0.91-1.53
	**35-44**	**0.01**	**1.40**	**1.08-1.82**	**0.03**	**1.34**	**1.03-1.75**
	**44-59**	**0.00**	**1.46**	**1.14-1.88**	**0.02**	**1.36**	**1.06-1.77**
	≥ 60	0.29	1.17	0.87-1.56	0.66	1.07	0.79-1.46
Educational level							
	Superior and higher		1.00		-	-	-
	High school	0.19	0.89	0.74-1.07	-	-	-
	Primary school	0.10	0.84	0.67-1.03	-	-	-
	No formal education	0.10	0.84	0.51-2.01	-	-	-
	Did not wish to answer	0.64	0.94	0.73-1.21	-	-	-
Income (minimum wages)^a^							
	More than 10		1.00		-	-	-
	More than 5 to 10	0.70	1.17	0.54-2.76	-	-	-
	More than 2 to 5	0.95	1.03	0.52-2.25	-	-	-
	From 1 to 2	0.98	0.99	0.51-2.16	-	-	-
	Less than 1	0.78	0.90	0.46-1.98	-	-	-
	Did not wish to answer	0.64	0.85	0.44-1.84	-	-	-
Religion^b^							
	Other religions or no religion		1.00			1.00	
	**Evangelical Christian**	**0.00**	**1.35**	**1.17-1.56**	**0.00**	**1.32**	**1.14-1.53**
	Comorbidity^c^						
	No		1.00			1.00	
	Yes	0.00	1.25	1.08-1.44	0.01	1.25	1.07-1.47
Living with someone with comorbidity^c^							
	No		1.00			1.00	
	Yes	0.09	1.14	0.98-1.32	0.58	1.05	0.88-1.25
Living with someone > 60 years							
	No		1.00		-	-	-
	Yes	0.46	1.08	0.87-1.34	-	-	-
Covid-19 vaccination status							
	More than two vaccine doses		1.00			1.00	
	**Up to two vaccine doses**	**0.00**	**1.31**	**1.11-1.54**	**0.01**	**1.30**	**1.06-1.59**
	Unvaccinated	0.18	1.60	0.76-3.04	0.69	0.81	0.24-2.03
Living with someone with comorbidity: Covid-19 vaccination status							
	More than two vaccine doses	-	-	-		1.00	
	Up to two vaccine doses				0.42	1.16	0.80-1.67
	**Unvaccinated**	**-**	**-**	**-**	**0.00**	**10.34**	**2.27-53.48**

OR: odds ratio; Note: boldface indicates statistical significance (p < 0.05).

a Refers to the Brazilian minimum monthly wage in force in 2022 of R$ 1,212.00 or US$ 233.53 (values based on exchange rate on 12/01/2022).

b Participants could select more than one religion.

c Includes obesity, *diabetes mellitus*, heart disease, high blood pressure, respiratory disease, cancer, hematological disease, advanced chronic kidney disease, chromosomal disorders with immune fragility, liver disease, autoimmune diseases, or immunodeficiencies.

Table 5 presents sensitivity analyses examining the robustness of associations between socio-demographic/clinical characteristics and the use of chloroquine, hydroxychloroquine, or ivermectin for Covid-19 prevention. When excluding participants who answered "sometimes" to the outcome question, higher odds of use were observed among participants living in Salvador (OR = 1.69; 95%CI 1.37-2.08), those self-identifying as Brown (OR = 1.37; 95%CI 1.06-1.78), aged 35-44 years (OR = 1.47; 95%CI 1.09-2.00) or 44-59 years (OR = 1.53; 95%CI 1.15-2.06), evangelical Christians (OR = 1.35; 95%CI 1.15-1.58), and individuals with comorbidities (OR = 1.35; 95%CI 1.13-1.60).

**Table 5:** Sensitivity analyses of factors associated with use of chloroquine, hydroxychloroquine, and ivermectin to prevent Covid-19: models excluding "sometimes" as a response category and stratified by city.

Socio demographic and clinical characteristics		Without "sometimes" as a category in outcome			Rio de Janeiro			Salvador		
		p-value	OR	95%CI	p-value	OR	95%CI	p-value	OR	95%CI
City										
	Rio de Janeiro		1.00		-	-	-	-	-	-
	**Salvador**	**0.00**	**1.69**	**1.37-2.08**	**-**	**-**	**-**	**-**	**-**	**-**
Race/skin color										
	White		1.00		-	-	-		1.00	
	Black	0.75	1.05	0.8-1.39	-	-	-	0.54	1.11	0.81-1.55
	**Brown**	**0.02**	**1.37**	**1.06-1.78**	**-**	**-**	**-**	**0.04**	**1.40**	**1.03-1.95**
	Asian	0.73	1.15	0.47-2.46	-	-	-	0.77	1.14	0.42-2.63
	Indigenous	0.67	0.64	0.04-3.22	-	-	-	0.62	0.59	0.03-3.02
Age group (years)										
	18-24		1.00			1.00		-	-	-
	25-34	0.08	1.30	0.97-1.77	0.13	1.52	0.89-2.66	-	-	-
	**35-44**	**0.01**	**1.47**	**1.09-2.00**	**0.02**	**1.87**	**1.11-3.24**	**-**	**-**	**-**
	**44-59**	**0.00**	**1.53**	**1.15-2.06**	**0.02**	**1.82**	**1.1-3.12**	**-**	**-**	**-**
	≥ 60	0.35	1.18	0.84-1.67	0.62	1.17	0.63-2.17	-	-	-
Religion^a^										
	Other religions or no religion		1.00			1.00			1.00	
	**Evangelical Christian**	**0.00**	**1.35**	**1.15-1.58**	**0.04**	**1.37**	**1.01-1.84**	**0.00**	**1.33**	**1.13-1.57**
Comorbidity^b^										
	No		1.00		-	-	-		1.00	
	**Yes**	**0.00**	**1.35**	**1.13-1.60**	**-**	**-**	**-**	**0.00**	**1.30**	**1.1-1.54**
Living with someone with comorbidity^b^										
	No		1.00		-	-	-		1.00	
	Yes	0.45	1.08	0.89-1.30	-	-	-	0.96	0.99	0.81-1.21
Covid-19 vaccination status										
	More than two vaccine doses		1.00		-	-	-		1.00	
	Up to two vaccine doses	0.14	1.19	0.94-1.49	-	-	-	0.04	1.28	1.01-1.60
	Unvaccinated	0.96	1.03	0.31-2.59	-	-	-	0.86	1.10	0.32-2.86
Living with someone with comorbidity: Covid-19 vaccination status										
	More than two vaccine doses		1.00		-	-	-		1.00	
	Up to two vaccine doses	0.49	1.16	0.76-1.73	-	-	-	0.41	1.20	0.78-1.83
	**Unvaccinated**	**0.02**	**6.99**	**1.3-39.23**	**-**	**-**	**-**	**0.05**	**6.53**	**1.00-49.88**

OR: odds ratio; 95%CI: 95% confidence interval;

Note: boldface indicates statistical significance (p < 0.05).

a Participants could select more than one religion.

b Obesity, *diabetes mellitus*, heart disease, high blood pressure, respiratory disease, cancer, hematological disease, chronic kidney disease at an advanced stage, chromosomal disorders with immune fragility, liver disease, autoimmune diseases or immunodeficiencies.

In stratified analyses, in Rio de Janeiro, higher odds were found among participants aged 35-44 years (OR = 1.87; 95%CI 1.11-3.24) and 44-59 years (OR = 1.82; 95%CI 1.10-3.12), and evangelical Christians (OR = 1.37; 95%CI 1.01-1.84). In Salvador, positive associations were seen for being Brown (OR = 1.40; 95%CI 1.03-1.95), evangelical Christians (OR = 1.33; 95%CI 1.13-1.57), having comorbidities (OR = 1.30; 95%CI 1.10-1.54), and having received up to two vaccine doses (OR = 1.28; 95%CI 1.01-1.60). Across both cities, being unvaccinated and living with someone with comorbidities was strongly associated with use (Rio de Janeiro: OR = 6.99; 95%CI 1.30-39.23; Salvador: OR = 6.53; 95%CI 1.00-49.88).

## DISCUSSION

In this study, factors associated with the use of chloroquine, hydroxychloroquine, and ivermectin to prevent Covid-19 were assessed in populations under high socioeconomic vulnerability in Brazil. A higher prevalence of chloroquine, hydroxychloroquine, and ivermectin use was observed in this study compared to low- and middle-income countries^17,22-24^, but was lower than that reported in earlier Brazilian studies conducted before the availability of Covid-19 vaccines (August-October 2020) in Mato Grosso (58%)^20^ and Manaus (31%)^3^. Local and national governments promotion of these drugs as part of the Covid-19 response could explain the high use of chloroquine, hydroxychloroquine, and ivermectin in Brazil and regional differences. The Brazilian federal government's role in misinformation was significant, including a Ministry of Health protocol recommending hydroxychloroquine for early treatment of Covid-19 based on symptoms alone, without laboratory confirmation, which persisted for more than a year^4^. The same document also conflated terminology by referring to "early treatment" as prophylactic or preventive^25^.

A collective stimulus to irrational medication use was promoted during the Covid-19 pandemic in Brazil, whether following medical prescriptions not based on evidence or via self-medication^26^. Although the Brazilian Federal Board of Medicine never promoted the use of chloroquine, hydroxychloroquine, and ivermectin as a preventive method, it played a significant role in supporting their efficacy as early treatment, authorizing physicians to prescribe these medications for early cases of Covid-19 under the principle of "physician autonomy"^4^. This decision to support prescriptions for early treatment encouraged many prescribers to adopt these drugs for prophylaxys^3,25^. The WHO termed this phenomenon an "infodemic," highlighting its negative impact on public health responses and on adherence to measures such as social distancing and vaccination^27^. Misinformation during the pandemic could have lasting effects, leading individuals to rely on unproven treatments and disregard scientific medical advice.

Consistent with the present findings, use of medications to prevent Covid-19 was associated with people aged 35-59 years in studies conducted in 12 Latin American countries^17^, as well as one in Mato Grosso, Brazil^20^. However, a study from Nigeria reported higher use among young people aged 20-29 compared to other age groups^22^. This indicates the need for continued education campaigns on the rational use of medication that focus on this age group in Brazil. Further investigation is needed to understand why this group appears particularly vulnerable. One hypothesis is that work-related pressures during the pandemic — especially among occupational groups required to remain active — may have increased the likelihood of using these drugs, potentially reinforced by social pressures within specific communities.

Anxiety and fear generated by Covid-19 may explain the increased use of ineffective prophylactic medications among people with comorbidities^28^. The association with evangelical Christians underscores the need for culturally sensitive health communication strategies. One tentative explanation of this result is that religiosity is strongly positively correlated with trust in information obtained from informal sources (spiritual leaders, family and friends, and websites of religious organizations), which are not necessarily reliable and may contradict scientific data^29^. Conversely, receiving more than two Covid-19 vaccine doses was associated with reduced use of ineffective preventive medications, suggesting that confidence in scientific methods can reduce irrational medication use. Dissemination of scientific information in clear terms and engagement of religious leaders and faith-based platforms may also enhance public understanding and acceptance of health measures.

In the study, unvaccinated individuals living with someone with comorbidities had higher odds of using chloroquine, hydroxychloroquine and/or ivermectin to prevent Covid-19. When living with someone with comorbidities, unvaccinated individuals might feel greater responsibility to adopt preventive measures — regardless of scientific validity — in an attempt to protect the vulnerable household member. The wide confidence intervals suggest that these results should be interpreted with caution, as they may reflect limited sample size or variability in this subgroup.

Sensitivity analyses support the robustness of the main findings. Excluding participants who responded "sometimes" did not substantially change the magnitude or direction of the associations observed in the main model. Stratification by city (Rio de Janeiro and Salvador) revealed broadly consistent patterns across locations, although some associations lost statistical significance, likely owing to reduced sample sizes in the stratified models. The concordance of results across these alternative specifications strengthens confidence in the validity of the main model, suggesting that the observed associations are not artifacts of outcome categorization or site-specific effects.

A key strength of this study is that data were collected during the Covid-19 pandemic and focused on an understudied vulnerable population prone to believing misinformation. However, use of a convenience sample from individuals seeking Covid-19 testing at PHC units may introduce selection bias. Additionally, overrepresentation of Salvador could limit the generalizability, as the associations may reflect local conditions or behaviors. Self-reported information on medication use may be subject to recall and social desirability bias, potentially leading to underreporting or overreporting of the outcome. Given its conceptual importance in capturing geographic variation in health communication and public policy, the variable "city" was retained in the final model despite elevated GVIF values, primarily owing to its interaction with income. While this decision aligns with the theoretical framework of the study, it introduces potential multicollinearity, which may have inflated standard errors and affected the precision of some estimates. Another limitation is that the questionnaire did not allow distinction of which specific medication(s) were being used by each participant. As a result, the outcome reflects combined use of any of these drugs, limiting the ability to assess patterns or determinants specific to each medication. Finally, categories with very low representation (*e.g.*, non-binary gender identity, no formal education) were retained in the models to preserve variable structure, resulting in wide confidence intervals for these estimates.

In conclusion, higher medication use for Covid-19 prevention in certain demographic groups highlights the need to address underlying health disparities. It is crucial to understand why these groups are more likely to use these medications and to ensure equitable access to reliable health information and resources.

## Data Availability

The data are available upon request.
